# Comparative analysis of closed versus open reduction and internal fixation in mandibular fractures: A prospective study

**DOI:** 10.6026/973206300222161

**Published:** 2026-04-30

**Authors:** S Sonal, Bibhu Prasad Mishra, Ashutosh Panda, Prabhat Kumar Soni, Kohinoor Acharya, Shree Mishra, Miral Mehta

**Affiliations:** 1 Department of Oral and Maxillofacial Surgery, ITS Dental College, Ghaziabad, Uttar Pradesh, India; 2 Department of Oral and Maxillofacial Surgery, Hi-Tech Dental College and Hospital, Bhubaneswar, Odisha, India; 3 Department of Oral and Maxillofacial Surgery, Guru Gobind Singh College of Dental Science and Research Centre, Burhanpur, Madhya Pradesh, India; 4 Department of Pediatric and Preventive Dentistry, Karnavati School of Dentistry, Karnavati University, Gandhinagar, Gujarat, India

**Keywords:** Mandibular fracture, closed reduction (CR), opens reduction, internal fixation, maxillomandibular fixation, miniplate osteosynthesis

## Abstract

Mandibular fractures are among the most frequently encountered maxillofacial injuries. Yet, the optimal management strategy between closed and open reduction with internal fixation (ORIF) remains a topic of considerable debate, given differences in functional outcomes and complication rates. Therefore, it is of interest to evaluate and compare the clinical, functional and radiographic outcomes of closed reduction (CR) using maxillomandibular fixation and ORIF using titanium miniplates in patients with isolated mandibular fractures. Eighty patients were randomly allocated into two groups-Group A (CR, n=40) treated with arch bar fixation for four to six weeks and Group B (ORIF, n=40) treated with titanium miniplates via intraoral or extraoral approaches - and followed for six months. The evaluated parameters included fracture union period, maximum mouth opening, masticatory efficiency, occlusal accuracy, complication rates, nutritional status and overall patient satisfaction. Thus, we show that while both methods achieve similar union and occlusal outcomes, ORIF provides superior functional rehabilitation and satisfaction, establishing it as the preferred choice when resources and expertise are available.

## Background:

The mandible, due to its high position and special arrangement of a horseshoe, as the sole mobile bone of the facial skeleton, is particularly susceptible to traumatic damage [1]. The second most frequent type of facial injury after nasal bone fracture is mandibular fractures (which comprise between 36-59 percent of all maxillofacial injuries in the different epidemiological reports in different parts of the world) [2]. The etiology of mandibular fractures varies across geographical regions and includes road traffic accidents, interpersonal violence, falls, sports injuries and industrial accidents, with road traffic accidents predominating in the developing world and interpersonal violence in most of the developed world [3]. The practical and aesthetic implications of mandibular fractures lie much further than the traumatic injury itself. The mandible forms the basis of mastication, deglutition, articulation of speech and the maintenance of the airways and also forms the basis of the facial form and expression [4]. Poor or improper management of mandibular fractures may lead to malocclusion, dysfunction of the temporomandibular joint, chronic pain, infection, malunion or non- union, facial deformity and high morbidity of a psychosocial nature [5]. Thus, the ability to choose the most effective treatment module that restores anatomical form, functional occlusion and mandibular biomechanics while minimizing complications remains the cornerstone of maxillofacial surgical practice. Traditionally, the most common treatment modality for mandibular fracture was closed reduction with maxillomandibular fixation, which has a history of over 100 years of success [6]. The fracture minimization of this technique is realized by using arch bars, eyelet wires, intermaxillary fixation screws and then a period of four to six weeks of mandibular immobilization to enable fracture healing [7]. The benefits of closed reduction include that it is relatively simple, does not involve a surgical incision, does not compromise the periosteal blood supply, is less expensive and can be performed where surgical facilities are inadequate [8]. The implementation of rigid and semi-rigid internal fixation in the 1970s and 1980s, which originated in work on compression plating and later developed further with miniplate systems, has radically altered the management paradigm for mandibular fractures [9].

The principles of open reduction and internal fixation include direct surgical exposure to the fracture location, anatomical reduction under direct visualization and fixation with titanium miniplates and monosortical screws in accordance with the principles of an ideally functioning stable fixation along the ideal lines of osteosynthesis as proposed by Champy [10]. Theoretical benefits of ORIF include accurate anatomical reconstruction, instantaneous recovery of mandibular function, no long-term impact, rapid restoration of oral alimentation, increased adherence to oral hygiene and enhanced patient compliance [11]. Although there have been decades of clinical experience with both modalities, the evidence base comparing them remains fragmented and inconclusive. Several retrospective studies have demonstrated better functional results with ORIF; however, these studies are prone to selection bias, as more complicated or displaced fractures are treated with open reduction [12]. Although they offer stronger evidence, randomized comparative studies have been few in number and have usually been confined to specific fracture types or to limited outcome measures [13]. A survey of systematic reviews identified a lack of high-quality prospective studies that could directly compare the two methods across clinically relevant outcome domains [14]. In addition, there exist crucial knowledge gaps regarding the comparability of these treatment modalities in terms of nutritional status in the recovery stage, restoration of masticatory efficiency and overall patient satisfaction in the long-term outcomes, which have steadily been cited as instrumental measures in trauma surgery [15]. The dietary restriction due to maxillomandibular fixation during closed reduction has been associated with critical weight loss, nutritional disorders and reduced quality of life. Still, these risks have not been strictly compared with the recovery process after ORIF in prospective studies [16]. Therefore, it is of interest to compare and assess the clinical, functional and radiographic outcomes of closed and open reduction and internal fixation in the treatment of isolated mandibular fractures, including fracture healing, functional recovery, complication profile, nutritional effects and patient satisfaction during a six-month follow-up period.

## Materials and Methods:

## Design and ethical background of the study:

It is a prospective, non-randomized, comparative clinical study conducted at the Department of Oral and Maxillofacial Surgery over a period of two years and forty-eight months.

## Sample size estimation:

The sample size of 36 patients in each treatment group was estimated based on published literature indicating that the mean difference in maximum mouth opening between the treatment groups was 5 mm, with pooled standard deviations of 7.5 mm and a two-sided significance level of 0.05. With an estimated 10% dropout rate, 50 patients were recruited in each group, yielding a study population of 80 patients.

## Selection and allocation of participants:

Eighty patients with diagnosed mandibular fractures who reported to the emergency department and outpatient clinic were enrolled. Treatment allocation was made according to clinical indications, fracture characteristics and patient factors, using a standardized decision protocol developed by a multidisciplinary group. Patients who had minimally displaced or favourable fractures that could be treated using either of the two treatment modalities were randomly assigned to treatment groups in an alternating manner to reduce selection bias.

## Inclusion criteria:

Age range of 18 to 60 years, isolated fractures of the mandible (single or double) confirmed clinically and radiographic imaging (Orthopantomograms and computed tomography) and presentation within seven days of injury, adequate dentition to apply arch bars (two teeth or more per quadrant in the fracture area) and desire to adhere to the follow-up schedule.

## Exclusion criteria:

Comminuted mandibular fractures, mandibular fractures with concomitant midface fractures (panfacial trauma), condylar fractures that needed surgical treatment, pathological fractures due to neoplastic or metabolic disease, fractures in edentulous or severely atrophic mandibles, patients with uncontrolled systemic diseases (diabetes mellitus with HbA1c > 8%), pregnant patients and fractures older than fourteen days at presentation.

## Group allocation:

[1] Group A - Closed Reduction (n=40): Erich arch bar and maxillomandibular fixation Fracture care.

[2] Group B - Open Reduction and Internal fixation (n=40): Fracture treatment by titanium miniplate osteosynthesis.

## Treatment protocols:

## Group A (closed reduction):

Erich arch bars were modified and placed on the maxillary and mandibular dental arches with 26-gauge stainless steel wires under local anesthesia with the addition of intravenous sedation or general anesthesia, as deemed necessary. Fracture reduction was done manually, directed by restoration of pre-injury occlusion and maxillomandibular fixation was done by fixing 24-gauge stainless steel wires. The fixation period was calculated based on clinical and radiographic evidence of fracture healing, which typically ranges from four to six weeks. Patients were equipped with wire cutters to use in case of airway obstruction to free themselves. The fixation period was prescribed to a liquid-soft blenderized diet. Guided jaw exercises were initiated as soon as fixation was released.

## Group B (open reduction and internal fixation):

Erich arch bars were used to provide temporary intraoperative guidance of reduction under general anesthesia with nasotracheal intubation. Body, parasymphysis and symphysis fractures were approached by an intraoral vestibular incision and angle fractures were approached by a submandibular (Risdon) incision when intraoral access was not available. A little elevation of the periosteum was done to expose the fracture area. This was performed under direct visualization to anatomically reduce, restoring occlusal relationships, which was confirmed by temporary maxillomandibular fixation. Fracture fixation was conducted with titanium miniplates (4 holes or 6 holes) of 2.0 mm and monosortical screws (2.0 mm diameter, 6 mm, -8 mm length) that were installed based on the principles of ideal osteosynthesis lines introduced by Champy. In Symphyseal and parasymphyseal fractures, two miniplates were utilized (one at the superior and the other at the inferior border). In angle fractures, one miniplate was used on the superior border of the external oblique ridge. After the plate fixation, the temporary maxillomandibular fixation was removed and occlusion was checked. Layers were used in wound closure. These patients had light-guiding elastics one to two weeks after surgery. Six weeks of progressive advancement were prescribed on a soft diet.

## Post-operative management:

Both groups were given standardized antibiotic prophylaxis (amoxicillin-clavulanate 625 mg three times/day for 5 days or clindamycin 300 mg three times/day in patients with penicillin allergy), analgesics (ibuprofen 400 mg three times/day with paracetamol 500 mg as needed) and chlorhexidine 0.12% mouthwash twice daily. According to the indication, tetanus prophylaxis was conducted.

## Outcome assessment:

Patients were assessed at regular intervals, including 1 week, 2 weeks, 4 weeks, 6 weeks, 3 months and 6 months after the operation.

## Primary outcomes:

[1] Fracture union: Evaluated clinically (lacks mobility, pain, or tenderness at the fracture site) and radiographically (presence of bridging callus and cortical continuity on orthopantomogram) at every follow-up visit. Union was categorized as: primary union (within 8 weeks), delayed union (816 weeks), or non-union (>16 weeks).

[2] Maximum mouth opening (MMO): Measured as maximum interincisal distance in millimeters with the use of digital calipers in every follow-up visit. Three measuring points were taken and averaged.

[3] Occlusal accuracy: Scale of four points: excellent (occlusion partially restored post-injury), good (slight disparity that does not need any intervention), fair (disparity that is noticeable and that demands slight occlusal adjustment) and poor (malocclusion of great magnitude and that warrants additional intervention).

## Secondary outcomes:

[1] Masticatory efficiency: Measured on a five-point scale by a standardized questionnaire that measures the power to chew foods of different consistency at 6 weeks, three months and six months.

[2] Complications: Documented and classified as: infection (wound infection, osteomyelitis), hardware-related (plate exposure, screw loosening, plate fracture), nerve injury (inferior alveolar nerve, marginal mandibular branch), malunion, non-union, tooth injury and wound dehiscence.

[3] Nutritional measure: The baseline, six-week and six-month records of body weight (kg) and body mass index (BMI).

[4] Patient satisfaction: measured 6 months using a validated 10-point Likert scale questionnaire on the functional recovery, facial appearance, experience throughout the treatment and overall satisfaction.

## Statistical analysis:

The SPSS software (version 26.0, IBM Corporation) has been used in data analysis. The Shapiro-Wilk test was used to check the normality. Continuous variables were presented as mean and standard deviation and the independent samples t-test or Mann-Whitney U test was used to compare them. Categorical variables were reported as frequencies and percentages and analyzed using the chi-square or Fisher's exact test. Longitudinal comparisons across groups were performed using repeated-measures ANOVA. Statistical significance was assessed using p-values <0.05.

## Results:

All eighty patients completed the minimum six-month follow-up period. The demographic and injury characteristics of the study population are presented alongside the results. The mean age was 31.4 ± 9.8 years, with a male predominance of 82.5%. There were no statistically significant differences between the groups in age (p = 0.463), sex distribution (p = 0.618), etiology of fracture (p = 0.527), or fracture site distribution (p = 0.384). Primary fracture union was achieved in 95% of CR patients and 97.5% of ORIF patients, with no statistically significant difference between groups (p = 1.000). Two patients in the CR group experienced delayed union, while one patient in the ORIF group developed delayed union associated with a postoperative infection. No cases of non-union were observed in either group. Maximum mouth opening demonstrated statistically significant differences between groups at all follow-up intervals beyond six weeks. At six months, the ORIF group achieved a mean MMO of 43.72 ± 3.84 mm compared to 39.46 ± 4.62 mm in the CR group (p < 0.001). Occlusal outcomes were comparable, with excellent or good occlusal restoration achieved in 87.5% of CR patients and 92.5% of ORIF patients (p = 0.461) (Table 1 - see PDF). The overall complication rate was 27.5% in the CR group and 25.0% in the ORIF group, with no statistically significant difference (p = 0.799). However, the pattern of complications differed notably between groups. The CR group experienced higher rates of temporomandibular joint stiffness and gingival/periodontal complications related to arch bars and prolonged fixation. The ORIF group demonstrated a higher incidence of wound-related complications, including three cases of surgical site infection and two cases of plate exposure requiring hardware removal. Inferior alveolar nerve paresthesia was more prevalent in the ORIF group, through most cases resolved spontaneously within three months. One case of marginal mandibular nerve weakness occurred in the ORIF group following a submandibular approach (Table 2 - see PDF). Significant differences were observed in nutritional parameters between groups. The CR group experienced a mean weight loss of 4.68 ± 1.92 kg at six weeks compared to 1.84 ± 1.26 kg in the ORIF group (p < 0.001). By six months, the CR group had regained most of the lost weight but remained at a marginally lower mean BMI compared to the ORIF group. Masticatory efficiency scores demonstrated significantly faster recovery in the ORIF group at all assessment points. Overall patient satisfaction at six months was significantly higher in the ORIF group (8.24 ± 1.18 versus 6.58 ± 1.72 on the 10-point scale, p < 0.001) (Table 3 - see PDF).

## Discussion:

This proposed comparative study will provide insight into the extensive body of evidence on the superiority or inferiority of closed reduction and open reduction with internal fixation in the treatment of mandibular fractures across various clinically significant domains. Although the two treatment modalities yielded similar fracture union rates and acceptable occlusive, major variations were observed in functional recovery, complication patterns, nutritional effects and patient satisfaction. The fact that all the equivalent fracture union rates were recorded between the two groups proves that, as per the biological perspective, both closed and open methods offer sufficient conditions to mandibular fracture healing. This result is consistent with the known fact that mandibular fractures have a high healing capacity when properly reduced and adequately stabilized, regardless of whether maxillomandibular fixation or miniplate osteosynthesis is used [17]. Mean fracture union between the two groups of around 5-6 weeks is in line with the well- recorded fracture healing period of secondary bone in the mandible in cases where sufficient blood supply and stability conditions are taken care of [18]. The much higher maximum mouth opening that the ORIF group achieved over the follow-up period is one of the most clinically significant outcomes of the current study. This is due to the long period of mandibular immobilization inherent to closed reduction, which leads to severe muscular atrophy, fibrous adhesions in the masticatory muscles and temporomandibular joint capsule and disuse changes that all hamper the restoration of normal mandibular range of motion [19]. Other studies have also reported chronic limitations in mouth opening following maxillomandibular fixation and some patients have required months of specialized physiotherapy to achieve satisfactory functional recovery [20]. The ORIF method avoids this issue by enabling early, controlled mandibular activity that maintains muscle tone and promotes periarticular fibrosis. The analysis of the complication profile showed that the two methods differed noticeably in their morbidity trends, but neither approach was overall better. The greater prevalence of gingival and periodontal complications in the CR group can be explained by the established tissue response to the long-term presence of arch bars and the lack of oral and dental care in those days when the jaws were immobilized [21]. The presence of dental plaque that periodically surrounds the arch bar wires, coupled with the fact that effective oral hygiene cannot be conducted under wire fixation, results in an environment that promotes gingivitis, periodontal inflammation and enamel degradation near the areas of wire fixation [22]. On the other hand, the ORIF group also showed complications of the surgical procedure per se, such as hardware infection, exposure of plates, nerve damage and these are the nature of any open surgical intervention on the maxillofacial area [23]. Inferior alveolar nerve paresthesia was more frequent in the ORIF patients, with 12.5 patients ranked against 5 in the CR. This finding is consistent with published data showing that surgical interference, periosteal elevation and hardware positioning near the inferior alveolar nerve canal are associated with a real risk of direct or indirect nerve damage [24]. It is, however, necessary to note that the majority of paresthesia cases in this study resolved spontaneously, which is consistent with the neuropraxia nature of these cases reported in the literature [25].

The isolated case of marginal mandibular nerve weakness after the submandibular approach is indicative of the susceptibility of this branch of the nerve to extraoral surgical access, a complication widely reported to have an incidence of 1-7% [26]. The data on nutritional impact are among the strongest points in comparing these two methods. The average body weight loss of almost 5 kilograms over six weeks of fixation in the CR group indicates the extreme dietary constraints of maxillomandibular fixation, which limit patients to a liquid or semi-liquid diet [27]. This extent of weight loss, which amounts to about 7 per cent of the initial body weight, has been shown in earlier studies to have serious metabolic implications, including protein depletion, micronutrient deficiencies and delayed wound healing, which could negatively affect the recovery process [28]. The significantly reduced weight loss in the ORIF group indicates the ability of such patients to return to solid food within days of operation, thereby ensuring they are well nourished in terms of calories and nutrients during the healing process. The higher masticatory performance of the ORIF group at each evaluation period carries significant consequences for quality of life in the postoperative stage. This capability to start chewing relatively early following surgery not only helps maintain nutritional adequacy but also provides neuromuscular rehabilitative and proprioceptive inputs, which are necessary for the restoration of normal masticatory functioning [29]. The literature on functional outcome after management of mandibular fracture has consistently shown that early restoration of function is associated with improved long-term masticatory function and decreased temporomandibular joint morbidity [30]. The high patient satisfaction scores in the ORIF group across all analyzed areas are notable but should be considered in light of the fundamentally different treatment experiences. The maxillomandibular fixation is an extremely burdensome experience to patients, which causes problems in communication, eating, social seclusion, impaired oral health and a constant fear of airflow blockage [31]. They are all contributing to the decreased levels of satisfaction despite acceptable clinical results, demonstrating the need to implement patient-centered measures in the evaluation of treatment outcomes. These findings have several limitations that should be considered when interpreting them. Although patient allocation is not random because a standardized protocol is used, it introduces the risk of selection bias, depending on fracture type and patient factors. The six months following, although sufficient to evaluate fracture healing and functional recovery might not reflect long-term complications such as late hardware removal, chronic pain conditions, or progressive occlusal alteration [32]. Also, the study did not perform a cost-effectiveness analysis, which is a significant factor, especially in healthcare settings with limited resources, where the availability of surgical infrastructure and titanium equipment can affect treatment choices [33]. The evidence base for clinical decision-making in the management of mandibular fracture would be strengthened by future multicenter randomized controlled trials with longer follow-up and economic analyses [34].

## Conclusion:

Both closed reduction with maxillomandibular fixation and open reduction with internal fixation achieve high rates of fracture union and acceptable occlusal alignment in isolated mandibular fractures. However, ORIF demonstrates superior outcomes in terms of mouth opening, masticatory efficiency, nutritional maintenance and overall patient satisfaction, whereas CR minimizes infection risks and surgical morbidity. Thus, we show that treatment choice should be individualized, balancing fracture characteristics, patient factors and resource availability to ensure optimal clinical and functional recovery.
